# Prevalence and determinants of late first antenatal care initiation in western Ethiopia: findings from a multi-centered cross-sectional study

**DOI:** 10.3389/frph.2025.1551706

**Published:** 2025-03-17

**Authors:** Atitegeb Alebachew Amsalu, Alemayehu Worku Yalew, Awgichew Kifle Zemlak

**Affiliations:** ^1^ School of Public Health, College of Health Sciences and Medicine, Wolaita Sodo University, Wolaita Sodo, Ethiopia; ^2^ School of Public Health, College of Health Sciences and Medicine, Addis Ababa University, Addis Ababa, Ethiopia

**Keywords:** late initiation of antenatal care, pregnant women, Western Ethiopia, antenata care, reproductive age

## Abstract

**Background:**

Late initiation of antenatal care (ANC) continues to be a significant public health issue in sub-Saharan African countries, including Ethiopia. However, despite the high prevalence of late ANC initiation in Ethiopia, only a few studies have been conducted, particularly in developing regions, such as Bebishangul-Gumez. Therefore, this study aims to assess the prevalence of late initiation of the first ANC and associated factors among pregnant women in Western Ethiopia, 2023

**Methods:**

A facility-based cross-sectional study was conducted in Western Ethiopia from March 28, 2023 to April 30, 2023. We employed a systematic random sampling technique to select 427 participants. Data were collected using an electronic capture technique with open data kit (ODK), then, transported to XLS and exported to Stata version 17 software for analyses. Bivariate analysis was performed at significance level of *p*-value < 0.25 to select candidate variables for multivariable analysis. In the final model, factors with a *p*-value ≤ 0.05 were considered significantly associated with late initiation of ANC.

**Results:**

A total of 414 pregnant women participated, yielding a response rate of 96.9%. The prevalence of late first ANC booking in this study was 56.8% (95% CI: 51.9, 61.5). Multivariable analysis revealed that being a housewives (AOR = 2.09, 95% CI 1.09, 4.01), having education status below secondary school (AOR = 3.5, 95% CI: 1.9, 6.1), having an unplanned pregnancy (AOR = 3.01, 95% CI 1.31, 6.90), lack of advise on when to start ANC (AOR = 1.74, 95% CI (1.09, 2.79), and attending ANC at hospital reduce the odds of late initiation of ANC (AOR = 0.41, 95% CI, 0.23, 0.72) were factors significantly associated with the late initiation of ANC.

**Conclusion:**

The prevalence of late initiation of ANC was found to be high in the study area. Educational status below secondary school, unplanned pregnancy, lack of advice on when to start ANC, housewives and attending ANC at hospital were factors found to be significantly associated with the late initiation of ANC. Therefore, policies should be developed to increase support for female education, maintain women's empowerment initiatives through economic changes, expand family planning programs to decrease unplanned pregnancies, and increase awareness in the early initiation of ANC.

## Introduction

Antenatal care (ANC) refers to services provided to pregnant women to ensure the best possible health outcomes for both mother and fetus (1). The timing of the first ANC contact is crucial for women and their babies to receive the best care and quality health outcomes (2). Early ANC visits are crucial for the assessment of gestational age (GA), screening for genetic and congenital disorders, provision of folic acid supplementation to lower the risk of neural tube defects (NTD), and screening and treatment for iron deficiency anemia (IDA) and sexually transmitted infections (STI). Furthermore, it is essential to convey vital medical information about treatable pre-existing disorder, and to offer guidance on preventable lifestyle risks (3–5).

Ethiopia has implemented the new ANC eight-contact model, following the 2016 WHO recommendation, to decrease perinatal mortality and enhance pregnancy-related outcomes. It is advisable that the initial contact for ANC to be initiated within the first trimester (before12 weeks). Various nations have different definitions for the early initiation of ANC. For example, in the context of Ethiopia, late ANC is defined as when a woman starts attending ANC after 12 weeks of gestational age (6). However, the prevalence of late ANC booking and its associated factors differ by country, region, and population. Globally, only 43% of ANC visits are timely bookings; developed regions have 85%, sub-Saharan regions have 25%, and developing regions 45% (2). According to the Ethiopian Demographic Health Survey (EDHS) 2016 and 2019 mini-DHS, only 20% and 28% of women received their first ANC during the first trimester of pregnancy, respectively (7, 8). Other studies done in different parts of Ethiopia regarding late initiation of ANC show, 67 from Addis Ababa, Ambo, southwest Ethiopia, systematic Meta-analysis, Gondar and 68 Jimma, that the prevalence of late ANC service booking were 47%, 86.8%,71.2%,64%, 69 63.8%, and 48%, respectively (9–14). Thus, the prevalence of late ANC initiation is still high in Ethiopia.

Late initiation of ANC increases the risk of various adverse outcomes during pregnancy, birth, and postpartum (15, 16). It may hide or delay the detection of fetal and maternal health problems such as hypertension, diabetes, anemia, antepartum hemorrhage, preterm labor, low birth weight and intrauterine fetal death. In addition, women who book their ANC late and fail to take folic acid in the first trimester will develop congenital neural tube defects. Furthermore, pregnant women who do not get screened for syphilis and HIV in the early stages of pregnancy will pass the disease to the fetus (17, 18). Ultimately, this can result in maternal and perinatal morbidity and mortality (19). A strategy used to reduce maternal mortality is timely and high-quality antenatal care (20). To alleviate the problems related to attending late ANC at first contact, the Federal Ministry of Health of Ethiopia is trying to expand ANC recommendations to eight contacts and to promote early ANC by HEW (6). Many studies have identified several factors that influence the late initiation of ANC, including the knowledge of the timing of ANC, age, maternal and husband education, maternal and husband occupation, family size, distance from health facility, transport cost, media exposure, parity, gravidity, abortion, and still birth. However, little is known about late ANC and associated factors in Ethiopia, particularly in Assosa Town. Therefore, this study assessed the prevalence and associated factors of late ANC initiation among pregnant mothers in Assosa Town health facilities.

## Methods

### Study area, design, and period

A facility- based cross-sectional study was conducted from March 28 to April 30, 2023 in Western Ethiopia, Benishangul-Gumuz Region, which is 447 km from Addis Ababa. According to the 2022 population estimation, Assosa Town has a total population of 72,227, of whom 16,864 were in the reproductive age group (15–49 years). Two health centers and one general hospital had ANC services in the town during the study period (21).

### Study participant

All pregnant women in Assosa Town were the source population. All pregnant women who had ANC contact in the study health facilities and available at the time of data collection were the study population**.** All pregnant women who had ANC visit in Assosa Town public health facilities during the data collection period were eligible for inclusion. Pregnant women with unknown gestational age during their first ANC contact and incomplete medical records, clients attending ANC, and who came with referral from other health facilities of the study site with incomplete medical record were excluded from the study.

### Sample size and sampling techniques

#### Sample size determination

The sample size was determined using a single population proportion formula for the first objective. The prevalence of late first ANC initiation was obtained from a previous study conducted in Jimma Town (48%) (13), and a 95% confidence level and 5% margin of error were considered.

$$\begin{array}{cc} n_{0}=\frac{Z_{\frac{\alpha }{2}}^{2}P(1-P)}{d^{2}} & n={\left(1.96\right)}^{2}0.48(1-0.48)/{\left(0.05\right)}^{2}=383.5\approx 384 \end{array}$$

The final sample size for the first objective was 384; after adding 10% nonresponse rates, the total sample was 427.

*n*_0_ = minimum sample size of the study subject.

*Z* = the standard normal distribution curve for a 95% confidence interval value of 1.96

p1 = proportion which was 0.48 from the previous study

*d* = margin of error (maximum acceptable difference (0.05)

The sample size for 2nd objective, the sample size was calculated using a double population formula using Epi info version 7 statical software, by considering factors that significantly associated with the outcome variable (Table 1).

**Table 1:** Sample size calculation for the second objective.

Variables (associated factors)	Confidence interval	Power	Percent in exposed	Adjusted OR	Sample size	Reference
Residence	95%	80%	12.2%	2.8	224	(36)
Educational status	95%	80%	77.6%	11.29	102	(41)
Intention to pregnancy	95%	80%	7.48%	0.142	92	(13)

The largest sample size is the one which is calculated for the primary objective is 427.

#### Sampling techniques

The study was conducted for a period of one month, during which the total number of pregnant women who received ANC services in the town was estimated based on, was the six moth report from Assosa Health Office. Accordingly, the number of pregnant women who had ANC visit for one month in the three health facilities was 794 (332 from Assosa Hospital, 246 from Assosa Health Center, and 216 from Sergalo Health Center) (21). Then, the sample was proportionally allocated to each health facility (number of monthly ANC clients). Therefore, Assosa Hospital took 177 of the sample, Assosa Health Center 132, and Sergalo Health Center 116 of the total sample calculated. Thus, dividing the total number of pregnant women by the sample size (*k* = *N*/*n* = 794/427) gave a sampling interval of 2. Therefore, the study subjects were selected from each health facility by systematic random sampling technique every 2 intervals. The first study participant was selected using the lottery method from the client registration log book as a sampling frame, and then pregnant women were enrolled in the study. For participants who could not remember their gestational age, their medical records were examined to gather additional information.

#### Data collection tool and procedures

Data were collected using a structured interviewer-administered and pre-tested questionnaire, which was developed based on the relevant literature (7, 22–24). Pre-test was done at Addis Ababa Lideta health enters. The questionnaire contained questions, including sociodemographic characteristics, past obstetric factors, knowledge and perception of ANC, health services- related factors, and current pregnancy-related factors. The data were collected using ODK software by those midwives have taking training working in the health facilities under close supervision of the principal investigator. A total of three data collectors with trained midwifery profession were assigned to study areas with one midwife to one to each ANC follow-up providing health center. To facilitate data collection, supervisor was assigned to each health center and hospital. The timing of late ANC initiation was determined by reviewing mothers' medical records to ascertain the initiation period, supplemented by direct interviews to identify factors associated with late initiation.

### Study variables

Dependent variable: late ANC initiation.

The independent variables were socio-demographic factors, past obstetric-related factors, health services-related factors, knowledge and perception on timing of ANC.

Socio-demographic variables were maternal age, residence, marital status, education status of women, educational status of husband, monthly income, occupational status of women, occupational status of husband, family size, and media exposure. The obstetric factors were parity, gravity, birth order, intention to pregnancy, decision-making on current pregnancy, advice when to start ANC, recognition of pregnancy, still birth, abortion, previous pregnancy complications, and previous ANC follow-up.

Health service-related factors were satisfaction with ANC services, appreciation of ANC distance from the health facility transport cost and waiting time.

Knowledge and perception about time of ANC.

## Operational definitions

### Late ANC initiation

First ANC contacts after 12 weeks of gestational age.

### Knowledge

Respondents who scored greater or equal to the mean score of those knowledge measuring questions were categorized as having adequate knowledge, otherwise inadequate knowledge (25).

### Perception

Who scored greater or equal to the mean score of those perception measuring questions were categorized as having good perception, otherwise poor perception.

## Data quality assurance

The data collection tool was developed in the English language and then translated into the local Amharic language. Additionally, a pre-test was conducted on 5% (*n* = 22) of the sample out of the study area. The necessary amendments were made for any ambiguity and clarity of the data collection instrument. The data collectors and supervisor received one-day training on the purpose of the study, the ODK software, the data collection tool, data collection methods, and ethical concerns during data collection. The supervisor and principal investigator monitored the data collection process of the data collectors. The data collection process was checked daily for inconsistencies and errors before sending the server.

## Data management and analysis

The data collection template was prepared in Excel and changed to an XLS form using the Kobo toolbox. Then, deployed to mobile ODK collect app and downloaded for data collection from get blank form by data collectors. Then, the daily collected data were sent to the central server. Data was exported to Stata version 17 statistical software for coding and analysis. Descriptive statistics were summarized using frequency, percentages, mean, and standard deviation. The association between each variable and late initiation of ANC was assessed using bivariable binary logistic regression analysis. Variables with *p*-values of less than 0.25 were included in the multivariable binary logistic regression analysis. In the final model, factors with *p* ≤ 0.05 were declared to be significantly associated with late initiation of ANC. Adjusted odds ratios with 95% confidence intervals were reported. Multicollinearity was checked among the independent variables using the variance inflation factor (VIF). The model fitness was also checked using the Hosmer–Lemeshow's goodness of fit test. Thus, the *p*-value for the Hosmer – Lemeshow's test was 0.475.

## Results

### Sociodemographic characteristics of participants

A total of 414 (96.9% response rate) pregnant women participated in the study. Hundred sixty nine (40.8%) respondents were in the age range of 25–29 years with a mean age of 25.5 (±4.3) years. The majority of them, 408(98.6%) were married and 364 (87.9) of respondents were of urban residence. 202 (48.8%) were orthodox religion follower. About more than half 237 (57.3%) of pregnant women have attended below secondary school and 239 (57.7%) were housewives. One hundred seventy (41.1%) of husbands' occupations were government employees. More than two-thirds (303 (73.2%) of the participants had a family size of less than or equal to three. More than half of the participants (239; 239(57.7) had monthly income of more than 6,000 with sample mean monthly income 10,429.7 (±9,184.1) ETB (Table 2).

**Table 2:** Socio demographic characteristics of participants in western Ethiopia, from March 28, April 30, 2023 (*n* = 414).

Variable	Frequency	Percent%
Age		
15–19	18	4.4
20–24	156	37.7
25–29	169	40.8
30 and above	71	17.1
Religion		
Orthodox	202	48.8
Muslim	154	37.2
Others^a^	58	14.0
Residence		
Urban	364	87.9
Rural	50	12.1
Marital status		
Married	408	98.6
Others^b^	6	1.4
Maternal educational status		
No formal education	12	2.9
Below secondary school	237	57.3
Above secondary school	165	39.8
Husband educational		
No formal education	14	3.4
Below secondary school	185	44.7
Above secondary school	215	51.9
Maternal occupational status		
Government employee	79	19.1
Private worker	73	17.6
House-wife	239	57.7
Others^c^	23	5.6
Husband's occupational status		
Government employee	170	41.1
Merchant	134	32.4
Farmer	36	8.7
Daily labour	51	12.3
Others^d^	23	5.6
Family size		
≤3	303	73.2
≥4	111	26.8
Monthly income(ETB)		
<3,000	23	5.6
3,000–5,000	119	28.7
5,001–6,000	33	7.9
>6,000	239	57.7
Media exposure		
No	85	20.5
Yes	329	79.5
Distance from the health facility(minute)		
<60	402	97.1
≥60	12	2.9

^a^ Protestant, catholic.

^b^ Single, divorced, widowed.

^c^ Student = daily labour.

^d^ NGO, drivers, gold farm, student.

### Current pregnancy characteristics of pregnant women

More than half 233 (56.3%) of the participants were multigravida. The majority of them had planned to be pregnant 362 (87.4%). Twenty (4.8%) developed pregnancy-related complications for their current pregnancy. Two-thirds of them experienced vaginal bleeding and they have attended due to complications, which accounts 18 (90%). Two hundred fifty-eight (62.3%) of the pregnant women recognized their pregnancy as a missed period. More than half 238 (57.5%) women were advised to start ANC. One hundred forty-seven (61.8%) pregnant women got advice from health professionals about the need to have early ANC. Most, 379 (91.6%) women decided themselves to start ANC (Table 3).

**Table 3:** Current pregnancy characteristics of pregnant women in late initiation of ANC in western Ethiopia, on March 28, April 30, 2023 (*n* = 414).

Variable		Frequency	Percentage
Gravidity			
Multi gravida		233	56.3
Prim gravida		181	43.7
Parity			
Nulliparous		181	43.7
Parity 1 and above		233	56.3
Birth order			
1		180	43.5
2–3		181	43.7
4–6 and above		53	12.8
Intent of pregnancy			
Planned		362	87.4
Unplanned		52	12.6
Pregnancy-related complications for current pregnancy			
Yes		20	4.8
No		394	95.2
If yes, what type of complication	PIHTN	358	15
	Hyperemesis gravidus	1	25
	Vaginal bleeding		40
	GDM		5
	PIHTN, Hyperemesis gravidus	1	5
	Urinary infection	1	5
ANC attend due to complications			
Yes		18	90
No		2	10
Decision-maker for the ANC			
My self		379	91.6
Husband		32	7.7
Others^a^		3	0.7
Means of recognizing pregnancy			
By urine test		156	37.7
Missed period		258	62.3
Advise when to start ANC			
No		176	42.5
Yes		238	57.5
Source of advice			
Husband		46	19.3
Health professionals		147	61.8
Friends		21	8.8
Others^b^		24	10.1

^a^ Both.

^b^ Other families, neighbours.

### Past obstetric characteristics of ANC

Approximately 221 (94.9%) pregnant women had an ANC visit during their previous pregnancy. One hundred twenty (59.7%) of them started ANC follow-up after 12 weeks of gestation. Half of 119 (51.1%) had one alive child. Nineteen (8.2%) of the women had a history of complications for previous pregnancy. Thirty-two (13.73%), sixteen (6.9%), and 13 (5.6%) of them had abortions, stillbirths, and child deaths, respectively. Some of them (8.6%) had a previous history of cesarean section. Seven (3%) women delivered at home (Table 4).

**Table 4:** Past obstetric characteristics of participants in late initiation of first ANC western Ethiopia, March 28, April 30, 2023 (*n* = 414).

Variable category	Frequency	Percentage
Abortion		
Yes	32	13.7
No	201	86.3
Still birth		
Yes	16	6.9
No	217	93.1
Child death		
Yes	13	5.6
No	220	94.4
No_alive children		
0	13	5.6
1	119	51.1
2–4	93	39.9
>4	8	3.4
Place of delivery		
Home	7	3
Health facility	226	97
Mode of delivery in a previous pregnancy		
Vaginal delivery	213	91.4
Cesarean/instrumental delivery	20	8.6
Time between births		
<2	48	20.6
≥2	185	79.4
Complications during the previous pregnancy		
Yes	19	8.2
No	214	91.6
ANC visit for a previous pregnancy		
Yes	221	94.9
No	12	5.2
Advise when to start ANC in the previous pregnancy		
Yes	182	78.1
No	51	21.9
Time of ANC for previous pregnancy		
Within 12 weeks of gestation	89	40.3
After 12 weeks of gestation	132	59.7

### Health services-related factors

Almost 219 (93.9%) respondents were satisfied with previous pregnancy ANC care. Among 251 (60.6) of the respondents attend health centers. Some 30 (12.8%) of the participants were getting the services after 2hrs of waiting (Table 5).

**Table 5:** Knowledge and perception of pregnant women regarding late initiation of antenatal care in western Ethiopia, March 28, April 30, 2023.

Variable	Frequency	Percentage
Knowledge in the first ANC initiation		
Adequate	117	28.3
Inadequate	297	71.7
Perception on the timing of ANC		
Good perception	243	58.7
Poor perception	171	41.3

### Knowledge and perception of pregnant women regarding the timing of antenatal care

Approximately 297 (71.74%) pregnant women had inadequate knowledge regarding first ANC initiation. More than half 243 (58.7%) of the respondents had a good perception of the timing of ANC (Table 6).

**Table 6:** Health- related factor of participants related to late initiation of first ANC in western Ethiopia, March 28, April 30, 2023 (*n* = 414).

Variable	Frequency	Percentage
Satisfaction with ANC in four previous pregnancy		
Yes	219	93.9
No	14	6.0
Welcomed appreciation of the ANC		
Yes	203	87.1
No	30	12.9
Waiting time (minute)		
<30	56	24.3
30–60	96	41.2
61–120	51	21.9
>120	30	12.9
Types of health facility attending		
Health center	251	60.6
Hospital	163	39.4
Transport cost of the health facility		
Pay	316	76.3
Not pay	98	23.7

### Prevalence of late first ANC contact among participants

**Figure 1:**
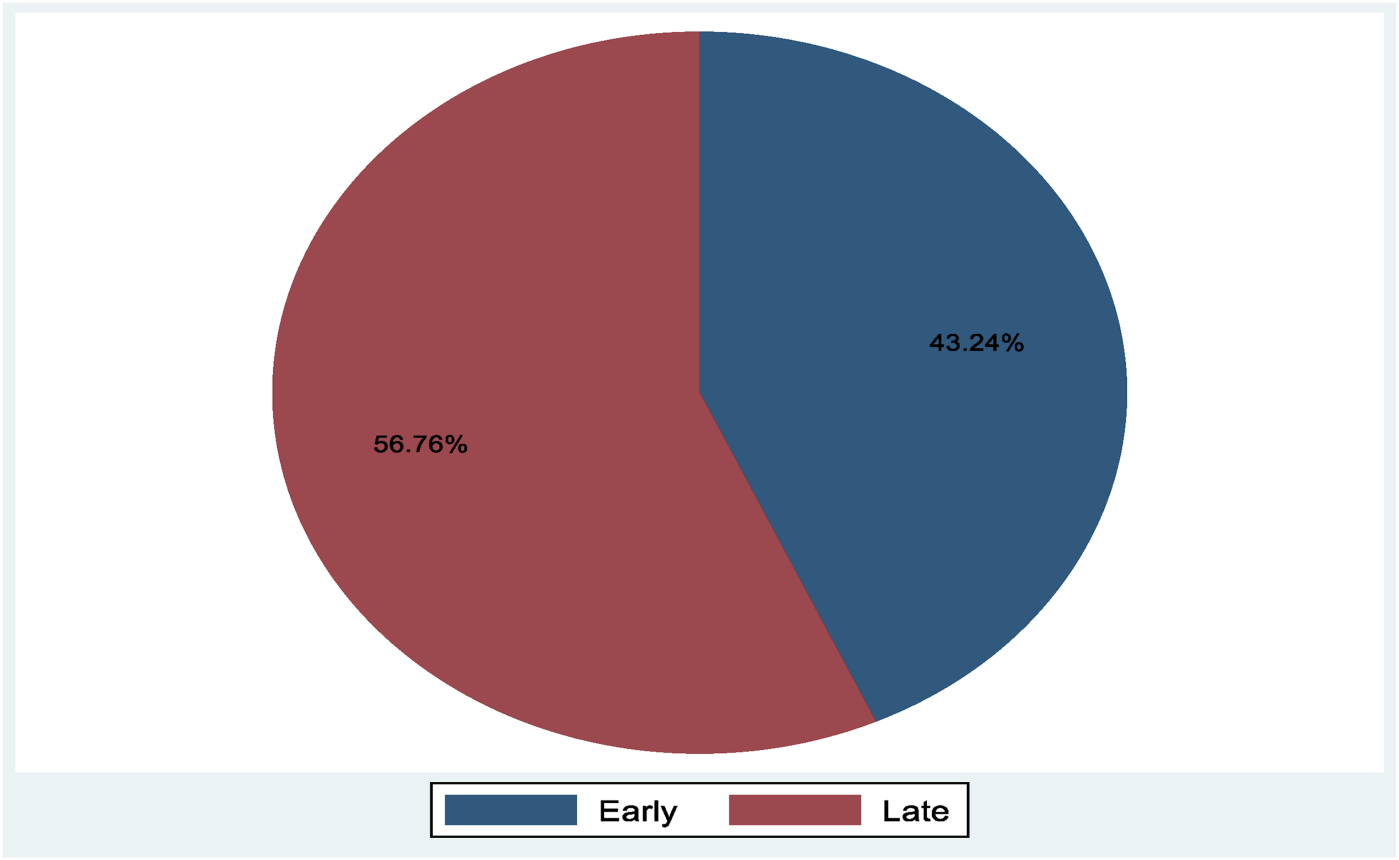
Prevalence of late first initiation of antenatal care for pregnant womens in western Ethiopia, March 28, to April 30, 2023.

Approximately, 179 (43.2%; 95% CI: 38.5, 48.1) mothers started their first ANC visit timely, and 235 (56.8%; 95% CI: 51.9, 61.5) mothers initiated ANC late (Figure 1).

### Factors associated with late initiation of the first ANC

Bivariate and multi-variable logistic regression analysis of the assessment of late initiation of first ANC (*n* = 414).

Variables with *p*-values less than 0.25 in the bivariate analysis, namely, age, marital status, residence, maternal education status, maternal occupation, husband occupation, family size, monthly income, media exposure, gravidity, parity, intent of pregnancy, birth order, advice when to start ANC, and type of health facility were entered into the multivariable analysis**.** After adjusting for other variables in the model, the odds of late ANC presentation among women with below secondary level education was 3.5 times higher compared to those with secondary education or higher (AOR = 3.5, 95% CI,1.9, 6.1), and the odds of housewives were two times higher compared with private workers (AOR = 2.0, 95% CI, 1.1,4.0). Similarly, unplanned pregnancy was three times at risk of late initiation of ANC compared with planned pregnancy (AOR = 3.0, 95% CI 1.3, 6.9). Mothers who did not receive advice on when to start antenatal care (ANC) were 1.7 times more likely to initiate ANC late compared to those who did receive such advice (AOR = 1.7, 95% CI 1.1, 2.8). In contrast to those attending the hospital, those women attending ANC at hospital had 60% reduction in the odds of late ANC start compared to those women who were attending ANC at the health centers (Table 7).

**Table 7:** Bivariate and multi-variable logistic regression analysis of the assessment of late initiation of the first ANC in western Ethiopia, March 28 to April 30, 2023 (*n* = 414).

Variable	Categories	Time of first ANC		COR (95%CI)	AOR (95% CI)	*P*-value
		Early%	Late%			
Age	15–19	11 (61.11)	7 (38.89)	1	1	
	20–24	71 (45.51)	85 (54.49)	1.88 (0.69, 5.1)	2.70 (0.84, 8.72)	0.096
	25–29	71 (42.01)	98 (57.99)	2.16 (0.80, 5.87)	2.91 (0.85, 10)	0.088
	30 and above	26 (36.62)	45 (63.38)	2.71 (0.93, 7.87)	2.52 (0.62, 10.2)	0.197
Residence	Urban	162 (44.51)	202 (55.49)	1	1	
	Rural	17 (34.00)	33 (66.00)	1.55 (0.83, 2.89)	1.25 (0.54, 2.89)	0.599
Maternal education	No formal education	2 (16.67)	10 (83.33)	7.89 (1.67, 37.2)	2.94 (0.5, 16.95)	0.227
	below Secondary school	76 (32.07)	161 (67.9)	3.34 (2.20, 5.06)	3.45 (1.94, 6.1)	0.001*
	Above the secondary school	101 (61.21)	64 (38.79)	1	1	
Maternal occupation	Government employee	46 (58.23)	33 (41.77)	0.66 (0.35, 1.25)	1.41 (0.57, 3.45)	0.450
	Private worker	35 (47.95)	38 (52.05)	1	1	0.025
	House-wife	84 (35.15)	155 (64.85)	1.69 (0.99, 2.88)	2.09 (1.09, 4.01)	0.959
	Others^a^	14 (60.87)	9 (39.13)	0.59 (0.23, 1.53)	0.97 (0.32, 2.94)	
Husband occupation	Government employee	77 (45.29)	93 (54.71)	1	1	
	Merchant	66 (49.25)	68 (50.75)	0.85 (0.54, 1.34)	0.59 (0.33, 1.05)	0.077
	Farmer	6 (16.67)	30 (83.33)	4.13 (1.63, 10.46)	1.42 (0.45, 4.49)	0.543
	Daily labour	15 (29.41)	36 (70.59)	1.98 (1.01, 3.89)	1.07 (0.45, 2.56)	0.870
	Others^b^	15 (65.22)	8 (34.78)	0.44 (0.17, 1.09)	0.50 (0.17, 1.43)	0.199
Family size	≤3	141 (46.53	162 (53.47)	1	1	
	≥4	38 (34.23)	73 (65.77)	1.67 (1.06, 2.62	1.4 (0.69, 2.84)	0.344
Monthly income	<3,000	6 (26.09)	17 (73.91)	2.76 (1.05, 7.24)	1.21 (0.37, 3.99)	0.743
	3,000–5,000	47 (39.50)	72 (60.50)	1.49 (0.95, 2.33)	0.99 (0.54, 1.81)	0.988
	5,001–6,000	8 (24.24)	25 (75.76)	3.04 (1.32, 7.02)	2.15 (0.84, 5.50)	0.117
	>6,000	118 (49.37)	121 (50.63)	1	1	
Media exposure	Yes	152 (46.20)	177 (53.80)	1	1	0.473
	No	27 (31.76)	58 (68.24)	1.84 (1.11, 3.05)	1.004 (0.49, 2.02)	` 0.990
Gravidity	Prim gravida	91 (50.28)	90 (49.72)	1	1	
	Multigravida	88 (37.77)	141 (62.23)	1.66 (1.12, 2.46)	0.12 (.002, 5.99)	0.290
Parity	Nulliparous	93 (51.38)	88 (48.62)	0.55 (0.37, 0.82)	0.35 (0.028, 4.41)	0.419
	Parity 1 and above	86 (36.91)	147 (63.09)	1	1	
Birth order	1	91 (50.56)	89 (49.44)	0.42 (0.21, 0.81)	0.30 (0.01, 5.9)	0.434
	2–3	72 (39.78)	109 (60.22)	0.65 (0.34, 1.26)	1.02 (0.42, 2.51)	0.950
	4–6 and above	16 (30.19)	37 (69.81)	1	1	
Intent of pregnancy	Planned	169 (46.69)	193 (53.31)	1	1	
	Unplanned	10 (19.23)	42 (80.77)	3.67 (1.79, 7.55)	3.01 (1.31, 6.90)	0.009*
Advise when to start ANC	Yes	116 (48.74)	122 (51.26)	1	1	
	No	63 (35.80)	113 (64.20)	1.64 (0.66, 4.05)	1.74 (1.09, 2.79)	0.020*
Types of health facility attending	Health center	86 (34.26)	165 (65.74)	1	1	
	Hospital	93 (57.06)	70 (42.94)	0.392 (0.26, 0.58)	0.41 (.23, 0.72)	0.002*
Perception in timing of ANC	Good	115 (47.33)	128 (52.67)	1	1	
	Poor	64 (37.43)	107 (62.57)	1.50 (1.007, 2.23)	0.73 (0.411.29)	0.286

^a^ Student, daily Labour.

^b^ NGO, drivers, gold farm, student.

* Statically significance in AOR, CI confidence interval, AOR adjusted odds ratio, COR =crude odds ratio, 1 = reference.

## Discussion

In this study, the prevalence of late initiation of first ANC was 56.8% (95% CI: 51.9, 61.5). This finding is in line with studies conducted in different regions of Ethiopia, such as Gedo (58.5%), Woldya (59.5), South Gondar (52.5%), and Myanmar (56%) (14, 26–28). However, our finding is lower than studies done in Zambia (86%) (29), Nigeria (65%) (30) Tanzania (70.4%) (31), South Benin (71.4) (32), East Wollega (81.5%) (33), Ilu Ababur (71.2%) (34), Gondar (63.2%) (34) and Mizan Tepi (66%) (35). possible reasons might be the current national emphasis given to ANC and the huge amount of work being done to improve maternal health outcomes in the country. Furthermore, differences in sociodemographic, health policy implementation between study areas, time gaps between the studies, ANC knowledge, and availability to health facilities have improved in Ethiopia compared with the past (36).

The prevalence in this study is also lower than the reported prevalence in Ethiopian EDHS 2016 (80%). This discrepancy could be explained by EDHSs having a larger sample size, including remote areas, and the time interval between the two studies. The prevalence of late first antenatal care booking is also found to be higher than that in the studies done in Addis Ababa (47%), Debremarkos (34.4%), Jimma (48%) (9, 10, 13). The reason for this gap can be that the study area is remote, so there is sociodemographic difference, less media access for information, less availability of extension service and transportation cost and cutoff point of outcome variable include (12–16 weeks) of pregnancy counted as early first antenatal booking and poor service delivery can also be the reason. Late first antenatal care booking was strongly associated with the late recognition of unplanned pregnancy.

Pregnant mothers with unplanned pregnancy have higher odds of late first antenatal booking than their counterparts. This finding is consistent with studies reported from Kembata Tembaro (37), Addis Ababa (10), Arbaminch (16), Dilla (38) and Jimma (13) and South Gondar (27). This might be due to lack of support from spouses or relatives during unplanned pregnancy, which could make women less motivated to seek ANC at an early stage. Different sociodemographic characteristics such as income, decision-making behavior, and information when to start ANC follow-up could result in late first ANC compared with women with planned pregnancies (10).

Pregnant mothers who did not have advice on antenatal care had 1.7 times higher odds of booking their first ANC late than those who had ANC advice. This was consistent with studies conducted in Southwest Ethiopia, Bench Sheko, Dilla, and Halaba (22, 35, 38). This might be because when pregnant women are informed on the timing of ANC booking, they will attend ANC earlier and their awareness on the time of ANC booking also increases.

Maternal education below secondary school was another factor associated with late first antenatal care. Pregnant women whose educational status was below secondary school were 3.4 times more likely to book their first ANC than their counterparts. This finding was in agreement with studies conducted in southwest Ethiopia, Kembata Tembaro, and Arbaminch (16, 37). This might be mainly because less educated people are less likely to seek information about the importance of early ANC booking and do not easily appreciate the danger signs of pregnancy.

Pregnant women's occupational status was significantly associated with late ANC booking. In this study, housewives had higher odds of booking their first ANC late than private workers. This might be because housewives are too busy to have time for early antenatal booking. They were also less likely to receive information about the importance of early first ANC; the majority education status was below secondary school, and had low media exposure.

Mother's preference on type of health facility for ANC was also a factor for late booking of first ANC. In this study, mothers who attended ANC at the hospital were more likely to book their first ANC early compared to with those who attended at the health center. This agrees with the findings of the study conducted in Rwanda (39). It might be due to the fact that presence of good quality of services given in hospitals. In contrast to this study, a study from Gondar (40) reported that pregnant mothers who attended health centers were less likely to book their ANC late compared with those who attended hospitals. This might be due to poor service delivery, poor satisfaction of the client, and long waiting time to the health center compared with the hospital in this study.

### Limitations of the study

The study design was cross-sectional, and cause and effect relationships were not identified. Since the data were collected by health professionals, there is a possibility that participants provided socially desirable responses, which might affect the accuracy of the data.

## Conclusion

Prevalence of late initiation of first ANC was found to be high in the study area. According to this study, educational status of mothers below secondary school, housewife women, unplanned pregnancy, attending ANC at Health center, and lack of advice when to start ANC were significant factors for late initiation of first ANC contact. Identifying mothers who have risk factors for late initiation of ANC and intervening by creating awareness through health education will contribute to the improvement of newborn and maternal outcomes and better complication readiness and birth preparedness of women. Policies should be developed to increase support for female education, maintain women's empowerment initiatives through economic changes, expand family planning programs to decrease unplanned pregnancies, and increase awareness in the early initiation of ANC.

## Data Availability

The original contributions presented in the study are included in the article/Supplementary Material, further inquiries can be directed to the corresponding author.
